# Differential Growth Factor Adsorption to Calvarial Osteoblast-Secreted Extracellular Matrices Instructs Osteoblastic Behavior

**DOI:** 10.1371/journal.pone.0025990

**Published:** 2011-10-05

**Authors:** Archana Bhat, Simeon A. Boyadjiev, Craig W. Senders, J. Kent Leach

**Affiliations:** 1 Department of Biomedical Engineering, University of California Davis, Davis, California, United States of America; 2 Section of Genetics, Department of Pediatrics, University of California Davis School of Medicine, Sacramento, California, United States of America; 3 Department of Otolaryngology-Head and Neck Surgery, University of California Davis School of Medicine, Sacramento, California, United States of America; University of California, Merced, United States of America

## Abstract

Craniosynostosis (CS), the premature ossification of cranial sutures, is attributed to increased osteogenic potential of resident osteoblasts, yet the contribution of the surrounding extracellular matrix (ECM) on osteogenic differentiation is unclear. The osteoblast-secreted ECM provides binding sites for cellular adhesion and regulates the transport and signaling of osteoinductive factors secreted by the underlying dura mater. The binding affinity of each osteoinductive factor for the ECM may amplify or mute its relative effect, thus contributing to the rate of suture fusion. The purpose of this paper was to examine the role of ECM composition derived from calvarial osteoblasts on protein binding and its resultant effect on cell phenotype. We hypothesized that potent osteoinductive proteins present during sutural fusion (*e.g.*, bone morphogenetic protein-2 (BMP-2) and transforming growth factor beta-1 (TGF-β1)) would exhibit distinct differences in binding when exposed to ECMs generated by human calvarial osteoblasts from unaffected control individuals (CI) or CS patients. Decellularized ECMs produced by osteoblasts from CI or CS patients were incubated in the presence of BMP-2 or TGF-β1, and the affinity of each protein was analyzed. The contribution of ECM composition to protein binding was interrogated by enzymatically modulating proteoglycan content within the ECM. BMP-2 had a similar binding affinity for each ECM, while TGF-β1 had a greater affinity for ECMs produced by osteoblasts from CI compared to CS patients. Enzymatic treatment of ECMs reduced protein binding. CS osteoblasts cultured on enzymatically-treated ECMs secreted by osteoblasts from CI patients in the presence of BMP-2 exhibited impaired osteogenic differentiation compared to cells on untreated ECMs. These data demonstrate the importance of protein binding to cell-secreted ECMs and confirm that protein-ECM interactions have an important role in directing osteoblastic differentiation of calvarial osteoblasts.

## Introduction

Craniosynostosis (CS), the premature fusion of cranial sutures, is a congenital defect occurring in one in 2000 live births.[1], [2] Sutural ossification occurs at the osteogenic front of cranial sutures through direct (membranous) bone formation, and this process has been largely attributed to increased osteogenic potential of resident osteoblastic cells and mutations in a select number of genetic targets. Osteoblasts and progenitor cells derived from both leporine and human patients with craniosynostotic diseases exhibit increased osteogenic potential in culture.[3], [4], [5] At the genetic level, mutations in at least seven genes (*FGFR1*, *FGFR2*, *FGFR3*, *TWIST1*, *EFNB1*, *MSX2* and *RAB23*) result in enhanced ossification of the sutures in CS patients.[6] Most of these genes harbor gain-of-function mutations, while mutations in *TWIST*, an upstream repressor of *FGFR* (fibroblast growth factor receptor) genes, induces loss of function leading to constitutive overexpression of these genes. These data suggest that abnormalities in growth factor signaling via FGFR contribute to the rate of suture fusion.[7], [8].

In addition to the activity of resident cells, calvarial bone formation is directed by the presence and activity of various growth factors secreted by the underlying dura mater.[9], [10] Specifically, the dural production of growth factors such as transforming growth factor-beta 1 (TGF-β1) and basic fibroblast growth factor (FGF-2), as well as the absence of inhibitors of bone formation (*e.g.*, noggin) correlate to the timing and location of suture fusion in rodent and rabbit models.[11], [12] The identification of these molecules during development has led to the delivery of antibodies and inhibitors as a proposed treatment option to slow synostosis.[13] However, this approach suffers from short- and long-term difficulties due to challenges in timing and stability of delivering such large molecules to the defect site.

The extracellular matrix (ECM) plays a critical role in directing the behavior of surrounding bone cells by instructing various processes including cellular proliferation, differentiation, and regulating the transport and signaling of endogenous osteoinductive growth factors.[14], [15] While osteoblasts from craniosynostotic patients appear to possess enhanced osteogenic potential, the contribution of the surrounding ECM to regulating growth factor signaling to neighboring cells has been largely unexplored. Differences in ECM composition, particularly glycosaminoglycans and proteoglycans, may alter the osteogenic profile of responsive osteoblasts due to availability of binding sites for endogenous osteoinductive molecules.[16] Chondroitin sulfate proteoglycans such as biglycan and decorin are involved in regulating postnatal skeletal growth and osteogenic differentiation of mesenchymal stem cells (MSCs) and preosteoblasts in culture.[17] The treatment of cell-produced ECMs with heparanase and chondroitinase to cleave heparan sulfate and chondroitin sulfate glycosaminoglycans the proximal binding sites for osteoinductive proteins, resulted in increased osteogenic differentiation of MSCs in culture due to continued BMP signaling.[18], [19] In light of previous reports describing the role of proteoglycans on growth factor binding, the purpose of this paper was to examine the role of ECM composition derived from calvarial osteoblasts on osteoinductive protein binding and its resultant effect on cell phenotype.

We hypothesized that calvarial osteoblasts from patients diagnosed with CS secrete ECMs that differentially bind osteoinductive proteins compared to cells from unaffected control individuals (CI), and the resulting binding capacity would subsequently modulate the osteogenic potential of associated cells. To test this hypothesis, decellularized ECMs were produced by calvarial osteoblasts from CS patients or unaffected CI, TGF-β1 or BMP-2 was adsorbed to each ECM, and the affinity of each protein for the ECMs was quantified. These two osteoinductive molecules were selected due to their presence and role in contributing to sutural fusion.[12], [13] To further explore the interplay between growth factor-ECM interactions and osteogenic response, osteoblast-secreted ECMs were enzymatically modified to reduce heparan sulfate and chondroitin sulfate proteoglycans, after which the affinity of each growth factor was characterized and the resultant effect on osteogenesis was assessed.

## Results

### Differential binding of osteoinductive proteins to osteoblast-produced ECMs

**Figure 1:**
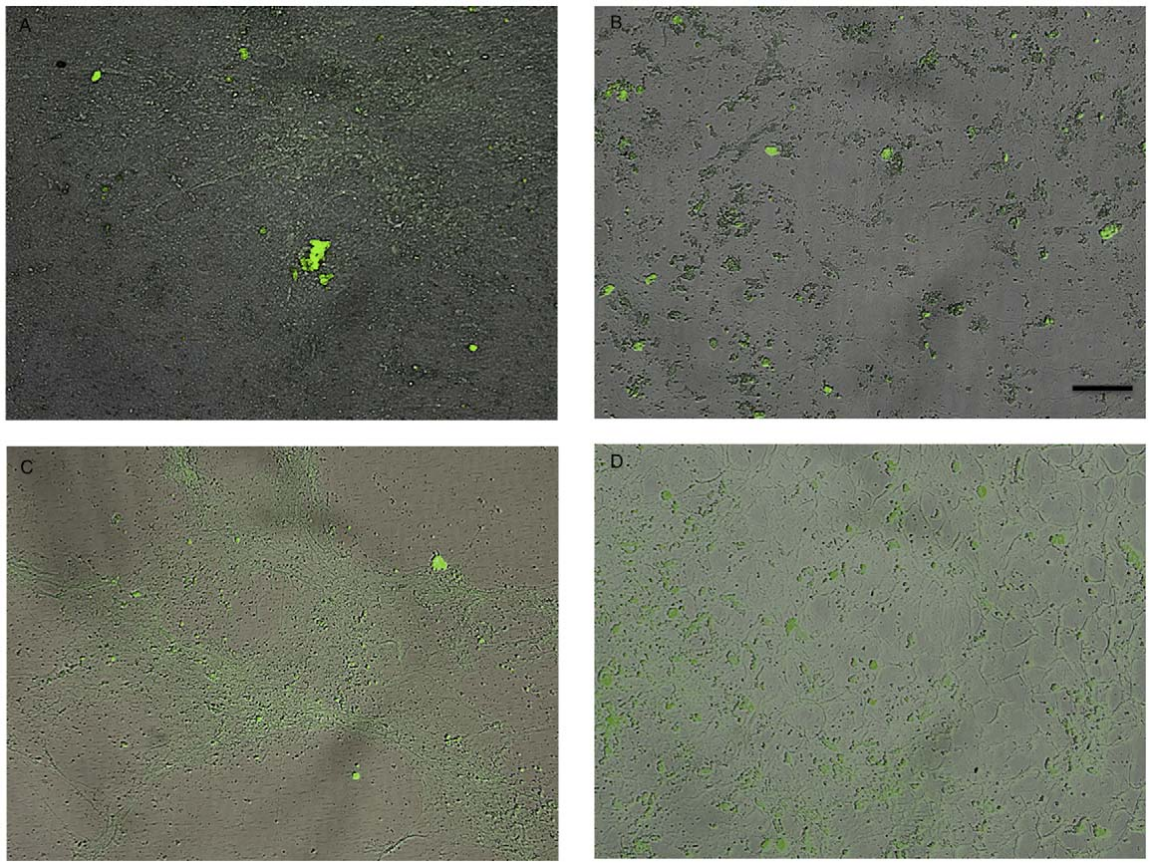
Overlay images (bright field and fluorescent) of TGF-β1 (0.05 ng/µl) (A, B) and BMP-2 (0.04 ng/µl) (C, D) bound to ECMs produced by CSObs (A, C) and CIObs (B, D). Images are representative of experiments performed in triplicate and are taken at 100x magnification; scale bars represent 50 µm.

**Figure 2:**
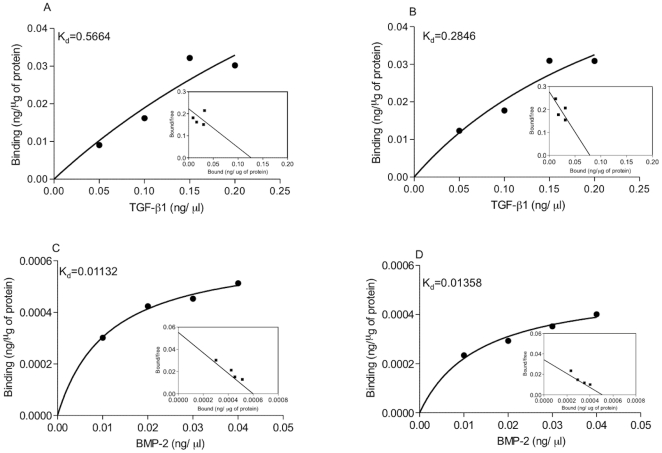
Scatchard plot analysis of TGF-β1 and BMP-2 binding to ECMs secreted by CSObs (A, C) and CIObs (B, D). Scatchard plot for representative data set in each culture condition is included as inset. Plots are representative of experiments performed in triplicate. K_d_ represents the dissociation constant.

We examined the adsorption of TGF-β1 and BMP-2 when exposed to ECMs secreted by calvarial osteoblasts derived from CI and CS patients by observing protein distribution and quantifying fluorescence. ECMs produced by CSObs contained significantly more total protein (1.15 ± 0.17 µg/µl) compared to ECMs secreted by CIObs (0.756 ± 0.02 µg/µl; *p*<0.01); hence, bound growth factor concentrations were normalized to total protein concentrations. Under fluorescence and brightfield microscopy, we observed greater ECM deposition by CSObs compared to ECMs secreted by CIObs, and proteins adsorbed to these ECMs with broad spatial distribution (**Fig. 1**). The dissociation constant (K_d_) for each protein was then calculated by quantifying the amount of free (unbound) protein and analyzed by a Scatchard plot. TGF-β1 maintained a significantly higher K_d_, and thus lower binding affinity in ECMs secreted by CSObs than CIObs (*p*<0.05) (**Fig. 2A, 2B**). No significant differences in BMP-2 binding were observed between the two ECMs (**Fig. 2C, 2D**). Importantly, BMP-2 exhibited substantially greater affinity for the ECMs, regardless of the cell source, compared to TGF-β1, as seen by the lower K_d_ values. We observed similar results when adsorbing growth factors to ECMs deposited at 21% O_2_ (*data not shown*), demonstrating that oxygen tension did not have a significant effect on the availability and composition of binding sites within these osteoblast-secreted ECMs.

### Characterization of ECM composition

**Figure 3:**
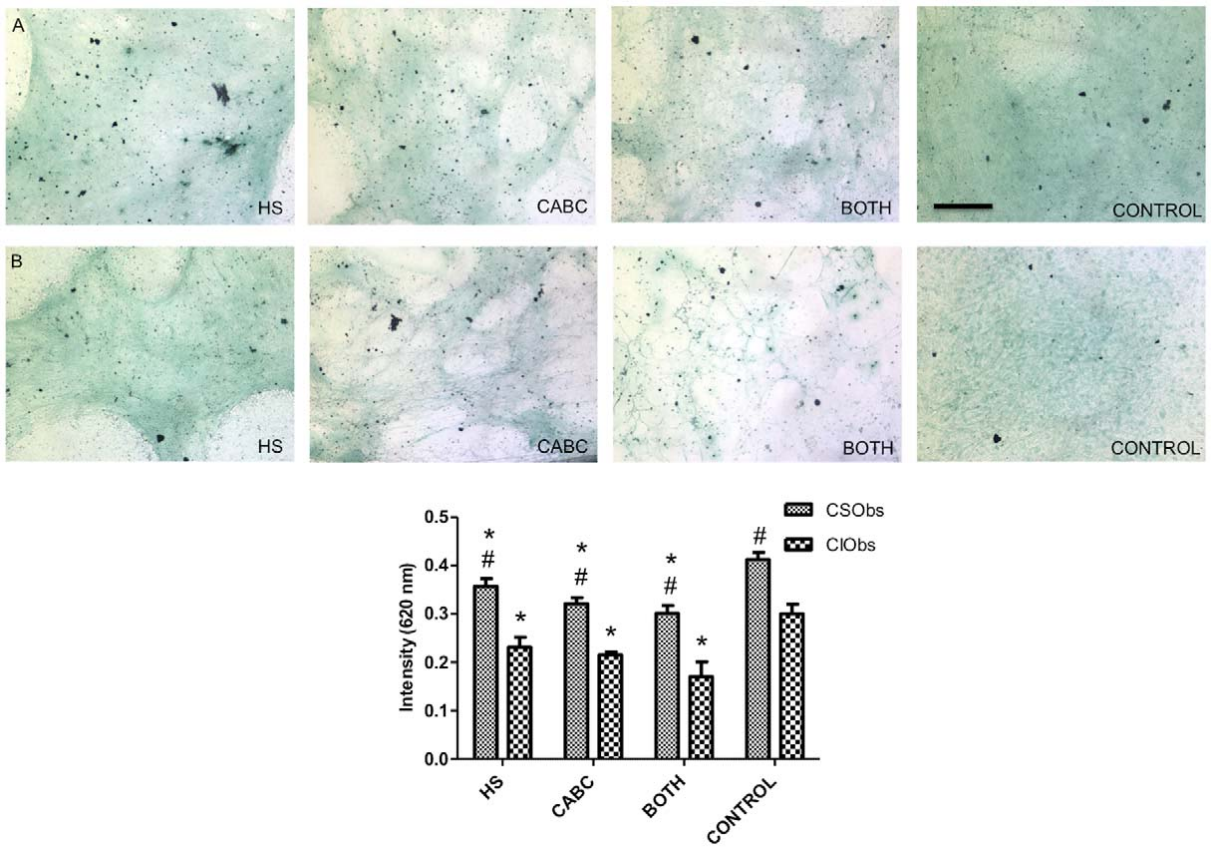
Alcian Blue stains of GAGs present in ECMs produced by CSObs (A) and CIObs (B) after heparanase (HS), chondroitinase ABC (CABC), both enzymes (BOTH) and without (CONTROL) enzyme treatment. Images taken at 100X magnification; scale bars represent 50 µm. C: Quantification of Alcian blue stain. *p<0.05 vs. control, #*p*<0.05 vs. CIObs ECM.

We examined the presence of cell-secreted mineral within decellularized osteoblast-secreted ECMs by Alizarin red staining to determine if differences in mineral content might contribute to differential protein binding. No evident staining was observed for ECMs produced by either osteoblast population *(data not shown)*. Next, we aimed to determine the effect of culturing cells in the presence of enzymes that interfere with GAG and proteoglycan content and binding to core proteins. Decellularized ECMs were stained with Alcian blue to qualitatively observe the effect of enzyme treatment on GAG content. Optical microscopy images confirmed increased Alcian blue staining in the untreated groups compared to ECMs grown in the presence of heparanase and/or chondroitinase ABC **(** **Fig. 3A, 3B** **)**. The qualitative findings were confirmed by quantification of dye intensity, with significantly lower GAG content in treated versus control (untreated) groups (**Fig. 3C** **)**. Although we failed to appreciate significant differences between ECMs treated with different enzymes, ECMs produced by CSObs consistently contained more GAG content compared to ECMs secreted by CIObs.

**Figure 4:**
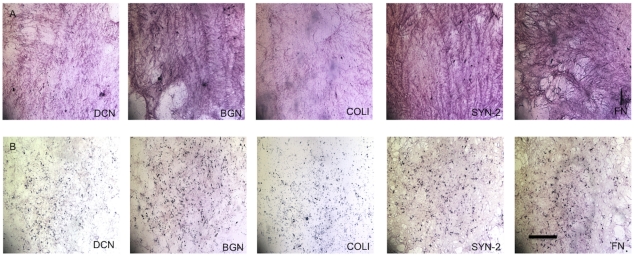
Immunohistochemical staining of molecules contained with ECMs secreted by CSObs (A) and CIObs (B). From left to right, images for decorin (DCN), biglycan (BGN), collagen type 1 (COLI), syndecan-2 (SYN-2), and fibronectin (FN). Images are taken at 40x magnification; scale bars represent 100 µm.

The presence of DCN, BGN, SYN-2, FN, and COL1 in enzyme-treated and untreated ECMs was examined with immunohistochemical staining **(** **Fig. 4** **)**. Although the different enzyme treatment groups resulted in similar staining intensities, ECMs secreted by CSObs exhibited substantially darker staining for DCN, BGN, SYN-2, COLI and FN compared to ECMs produced by CIObs for all groups.

### Enzyme-treated ECMs modulate osteogenic response of CSObs

**Figure 5:**
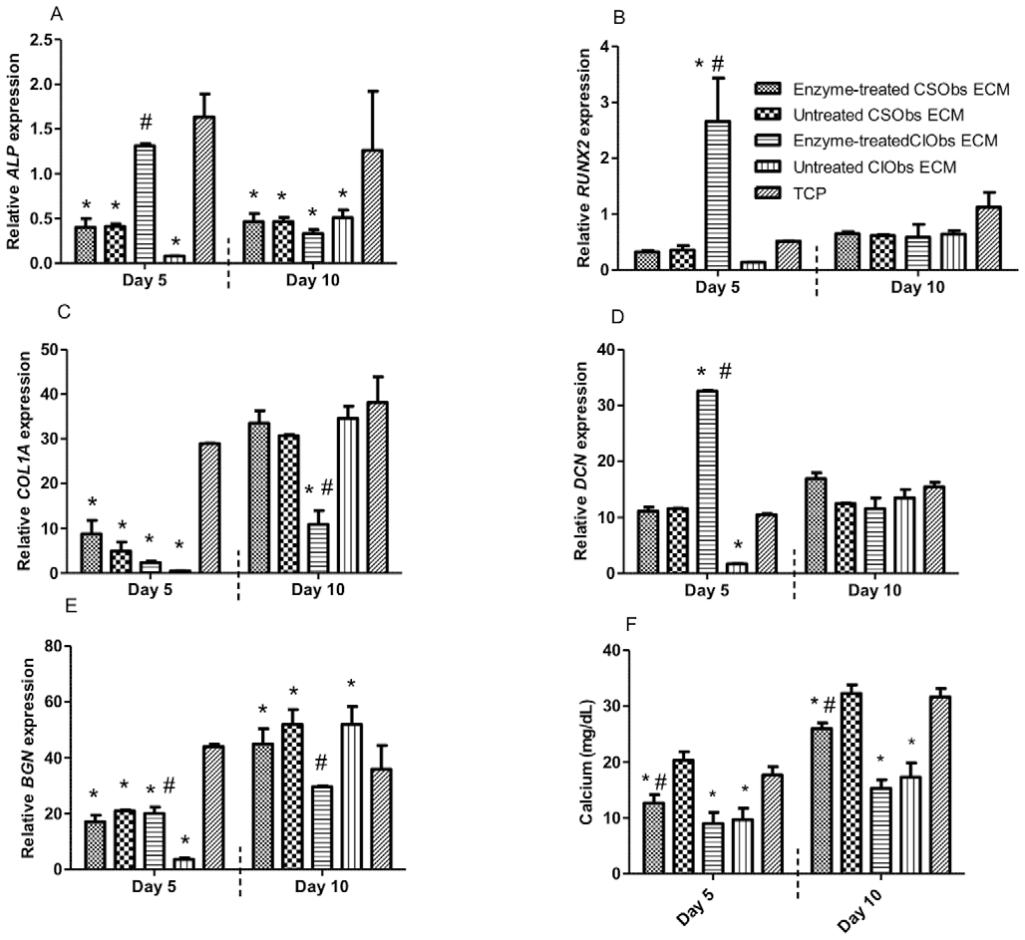
Quantitative PCR results for genes monitored in CSObs when seeded on enzyme- treated or untreated ECM from CSObs or CIObs or TCP: *ALP* (A), *RUNX2* (B), *COL1A* (C), *DCN* (D), *BGN* (E), and calcium deposition (F). Values reflect fold change in the target mRNA expression over RPL13. #*p*<0.05 vs. untreated ECM; *p<0.05 vs. TCP.

**Figure 6:**
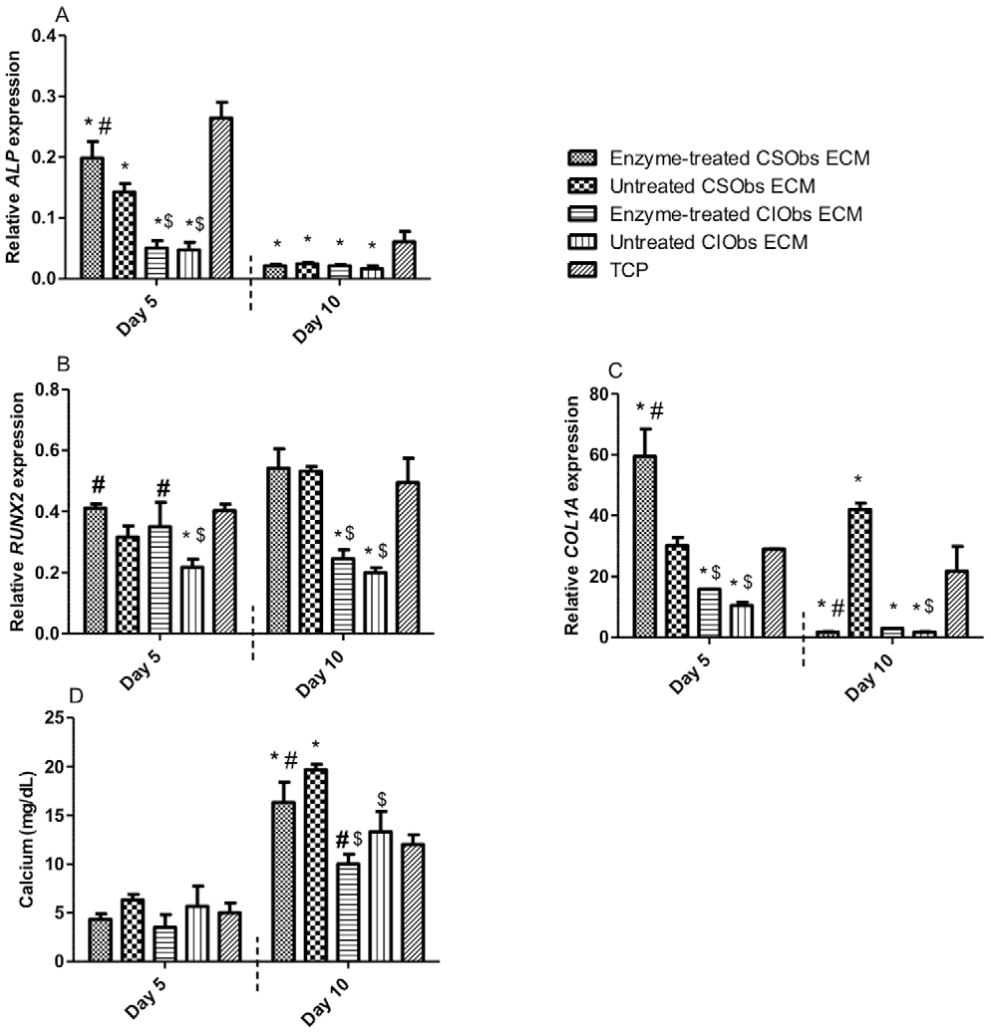
Quantitative PCR results for genes monitored in CIObs when seeded on enzyme- treated or untreated ECM from CSObs or CIObs or TCP: *ALP* (A), *RUNX2* (B), *COL1A* (C), and calcium deposition (D). Values reflect fold change in the target mRNA expression over RPL13. #*p*<0.05 vs. untreated ECM; *p<0.05 vs. TCP; $*p*<0.05 vs. CSObs ECM.

We used qPCR to measure the osteogenic response of CSObs when seeded on ECMs produced by CSObs or CIObs deposited in the presence of GAG-interfering enzymes (**Fig. 5**). We did not detect stark differences in gene expression for cells grown on ECMs in the presence of individual enzymes versus joint administration. Therefore, we elected to present only qPCR for cells seeded on ECMs deposited simultaneously with both heparanase and chondroitinase (enzyme-treated group). After 5 days, CSObs demonstrated significantly less *ALP* expression on all ECMs except the enzyme-treated CIObs ECM compared to untreated CIObs ECM and TCP, while culture for 10 days revealed consistent inhibition of *ALP* expression for all ECMs (**Fig. 5A**). We observed a similar pattern for both *RUNX2* (**Fig. 5B**) and *DCN* (**Fig. 5D** **)**. *COL1A* expression in CSObs was reduced for cells grown on all ECMs compared to TCP control after 5 days. However, after 10 days *COL1A* expression was reduced only in CSObs grown on enzyme-treated CIObs ECM compared to all other groups (**Fig. 5C**). We examined mRNA expression of *DCN* and *BGN* in reseeded CSObs to probe if cells would attempt to replenish an enzymatically-treated matrix. At 5 days, *BGN* mRNA levels were significantly higher in CSObs on enzyme-treated CIObs ECM compared to cells grown on untreated CIObs ECM, but mRNA levels were all significantly lower than cells on TCP (**Fig. 5E**). This response was not observed for CSObs seeded on cell-matched ECMs. However, *BGN* expression increased after 10 days for cells on untreated ECMs produced by CIObs versus enzyme-treated platforms. To determine if changes in gene expression altered the downstream osteogenic response, we quantified calcium deposition by CSObs cultured on ECMs secreted by CIObs versus native ECMs (**Fig. 5F**). After 5 and 10 days, CSObs cultured on ECMs secreted by CIObs ECM exhibited significantly lower amounts of deposited calcium compared to those cultured on native ECM, regardless of the enzyme treatment. When examining the behavior of CIObs on ECMs produced by CSObs, we observed significantly greater *ALP* expression in CIObs after 5 days compared to CIObs seeded on ECMs produced by CIObs (**Fig. 6A**). Similar trends were observed for *RUNX2* (**Fig. 6B**), *COLIA* (**Fig. 6C**), and calcium deposition (**Fig. 6D**) after 10 days, thus demonstrating the osteogenic potential of ECMs secreted by CSObs.

### Growth factor binding is altered by enzyme treatment of ECMs

The deposition of osteoblast-secreted ECMs in the presence of heparanase, CABC, or both enzymes resulted in significantly increased values of K_d_ for both TGF-β1 and BMP-2 as compared to untreated ECMs, confirming reduced protein binding for these matrices (**Table 1**). Furthermore, the application of both enzymes significantly reduced protein binding compared to ECMs produced by a singular enzyme. Similar to data collected without enzyme treatment (**Fig. 2A-B**), K_d_ was significantly higher and thus binding of TGF-β1 was lower for enzyme-treated ECMs deposited by CSObs compared to CIObs-secreted ECMs. We did not observe significant differences in BMP-2 binding between enzyme-treated ECMs deposited by either osteoblast population, perhaps due to the enzyme concentration used. However, BMP-2 binding was reduced on enzyme-treated ECMs compared to untreated ECMs, thus confirming the efficacy of removing protein binding sites during enzyme treatment.

**Table 1:** Dissociation constants (K_d_) for TGF-β1 and BMP-2 binding to enzyme-treated ECMs.

Enzyme treatment	TGF-β1 K_d_ (ng/µl)		BMP-2 K_d_ (ng/µl)	
	CIObs	CSObs	CIObs	CSObs
heparanase	0.566±0.05^#^	1.493±0.002^#^ *	0.256±0.004^#^	0.2663±0.021^#^
chondroitinase	0.644±0.04^#^	1.301±0.009^#^ *	0.268±0.039^#^	0.288±0.018^#^
Both enzymes	1.496±0.03	2.337±0.279*	0.454±0.043	0.460±0.044
None	0.459±0.07^#^	0.734±0.017^#^ *	0.0289±0.002^#^	0.024±0.002^#^

Values are represented as mean ± std. dev. (n = 3).

*p<0.05 vs. corresponding CIObs ECM, ^#^ *p*<0.05 vs. both enzymes.

### Differential binding of growth factors to enzyme-treated ECMs modulates osteogenic response

**Figure 7:**
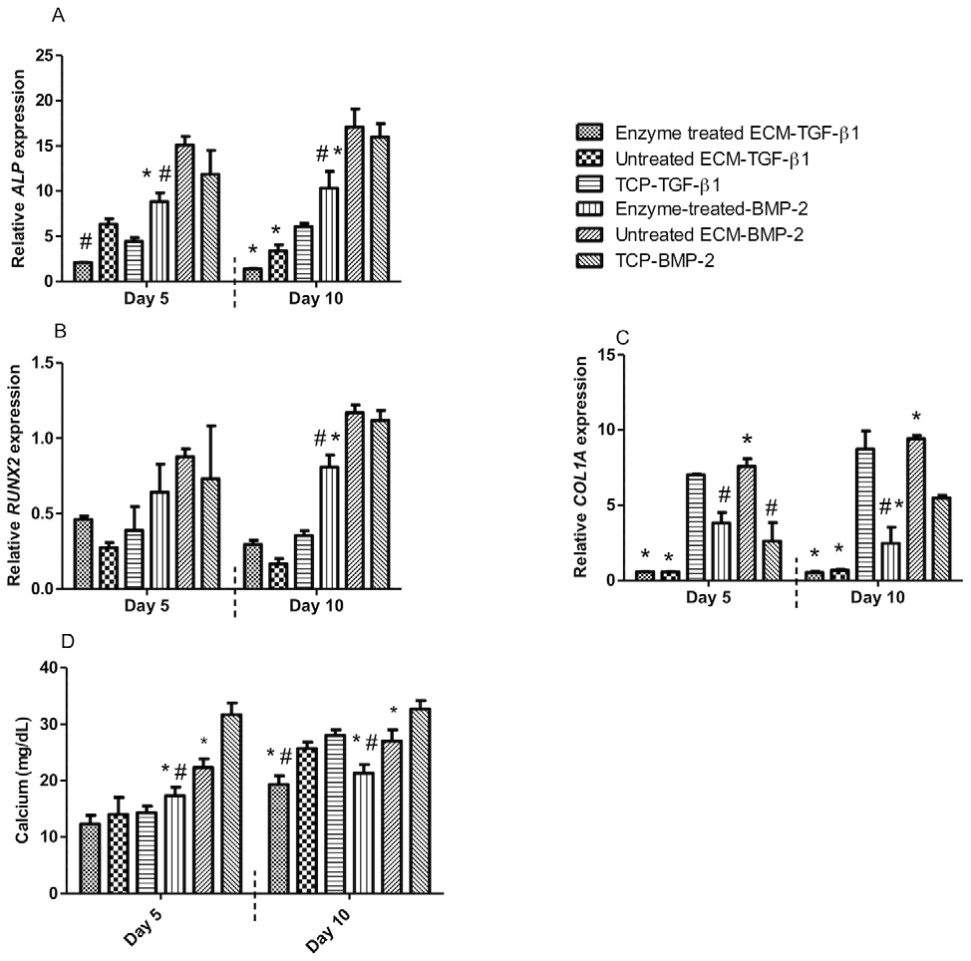
Quantitative PCR results for genes monitored in CSObs seeded on enzyme-treated or untreated ECM from CIObs or TCP control in the presence of TGF-β1 or BMP-2: *ALP* (A), *RUNX2* (B), *COL1A* (C), and calcium deposition (D). Values reflect fold change in the target mRNA expression over RPL13. #*p*<0.05 vs. untreated ECM, *p<0.05 vs. TCP.

After determining differences in the osteogenic response of cells seeded on ECMs and protein binding on enzyme-treated substrates, we examined the osteogenic response of CSObs when seeded on ECMs and cultured in the presence of TGF-β1 or BMP-2. In the absence of osteoinductive proteins, the osteogenic response of CSObs seeded on ECMs produced by CIObs was lower than those seeded on ECMs produced by CSObs. Thus, we characterized osteogenic potential of CSObs seeded on CIObs-produced ECM in the presence of either osteoinductive protein to detect greater differences due to each stimulus (**Fig. 7**). In the presence of exogenous TGF-β1, cells cultured on enzyme-treated ECM exhibited lower *ALP* expression after 5 and 10 days compared to cells on untreated ECM. Conversely, *RUNX2* expression was greater for cells on enzyme-treated ECMs with TGF-β1. Despite differential binding of TGF-β1 to enzyme-treated versus untreated ECMs, we did not detect differences in *COL1A* expression at 5 or 10 days, yet mRNA expression in both groups was significantly lower than cells cultured on TCP in the presence of TGF-β1. After 10 days, the amount of calcium deposited by cells seeded on enzyme-treated ECMs stimulated with TGF-β1 was significantly lower compared to cells on untreated ECMs. In the presence of BMP-2, the expression of each osteogenic gene was significantly lower in CSObs cultured on enzyme-treated ECMs compared to cells on untreated ECMs at 5 and 10 days. We detected a similar trend in calcium deposition at 5 and 10 days.

## Discussion

The ossification of developing human calvarial tissue is a complex process dependent upon the coordination of endogenous osteoinductive signaling cues and the osteogenic potential of responsive cells. Craniosynostosis, the premature fusion of the cranial sutures, restricts brain growth and requires surgical intervention to provide space for normal development. To investigate causality of this disease state, a significant portion of studies have focused on genetic mutations involving FGF receptors and DNA binding proteins such as MSX2 and TWIST [20]. In addition, others have shown differences in local TGF-β1 and insulin-like growth factor (IGF1) between patent and fused sutures [21], hence leading to recent therapeutic approaches of blocking the activity of these constituents with local antibody delivery. However, current treatment methods for craniosynostosis lack techniques to slow the increased osteogenic potential of native osteoblasts, and the contribution of the surrounding ECM is unclear. The results of this study demonstrate that cell-secreted ECMs exhibit differential interaction with osteoinductive cues, potentially providing a new target for treating patients diagnosed with craniosynostosis.

The interaction between cells and the surrounding ECM is critical to instruct cell phenotype, and these interactions have been examined *in vitro* and *in vivo* [22], [23]. The characterization of this behavior *in vitro* is commonly simplified to examining cell behavior on individual ECM proteins or on tissue culture plastic, yet the situation *in vivo* is more complicated, involving the interaction of cells with a complex array of proteins and polysaccharides. Thus, the use of cell-secreted decellularized ECMs provides a more physiologically relevant tool to explore the role of the ECM on cell behavior. We and others have demonstrated how decellularized ECMs can be used to control the cell fate process at different stages of differentiation.[14], [24] The results of our study confirm reduced binding of TGF-β1 to ECMs deposited by CSObs compared to CIObs. These results agree with previous studies where decreased TGF-β1 expression was detected in fused sagittal sutures compared to patent posterior frontal sutures.[25] Although the exact mechanism of TGF-β1 activity in bone formation is not clearly understood, TGF-β1 plays a major role in the bone remodeling process by regulating bone resorption.[26] Hence, reduced binding affinity of TGF-β1 to ECMs secreted by CSObs may contribute to increased bone formation seen in patients diagnosed with craniosynostosis.

Constituents of the ECM, particularly proteoglycans, play an important role in regulating the binding, transport, and signaling of various growth factors including TGF-β1 and BMP-2.[27] Upon analyzing the ECM secreted by various osteoblastic populations, others have reported differences in the expression of proteins including syndecan, decorin, and biglycan in normal osteoblasts versus those isolated from patients diagnosed with syndromic craniosynostosis.[16] The presence of biglycan and decorin has been closely associated with BMP-2 and TGF-β1 signaling, respectively.[28], [29] The increased amounts of decorin and biglycan in ECMs secreted by CSObs compared to CIObs likely represent a significant contribution to differences in growth factor binding to each ECM. This is in agreement with earlier studies demonstrating that the sulfated chondroitin sulfate structures on the surface of osteoblasts enhance osteogenic activity due to increased BMP-2 binding.[30] In addition to its effect on osteoblast activity, certain chondroitin sulfate units reduced osteoclast activity and impaired bone resorption.[31]

These data support previous results that demonstrate the osteogenic differentiation of cells of the osteoblastic lineage can be modulated in the presence of GAG-modifying enzymes.[17], [32] When cultured on enzyme-treated ECMs produced by CIObs for short periods, we observed that osteoblasts from craniosynostosis patients exhibited increased expression of *ALP* and *RUNX2*, both early indicators of osteogenesis, compared to cells cultured on untreated ECMs. This is consistent with reports of rare *RUNX2* duplications in CS patients.[33] Also, this increase in *ALP* at earlier time points could be attributed to the lower amount of *ALP* present in enzyme treated CIObs ECMs compared to the ECMs secreted by CSOBs. However, CSObs exhibited significant reductions in *COL1A* expression at 10 days when cultured on enzyme-treated substrates compared to untreated ECMs or TCP. Furthermore, increased decorin and biglycan within ECMs deposited by CSObs, even after enzyme treatment, may promote binding of endogenous BMP-2 compared to ECMs produced by normal osteoblasts, thus resulting in early increases in osteoblast activity. Despite significant increases in *ALP* and *RUNX2* expression, we did not detect differences in calcium deposition by CSObs on ECMs deposited by CIObs. These data suggest that CSObs are responsive to changes in their environment, suggesting that CSObs rapidly remodel the underlying matrix or lack necessary sensitivity to exhibit differences in mineral content. The temporal response of CSObs to an underlying ECM merits further investigation to fully understand how these cells remodel their surroundings and undergo osteogenic differentiation. We also observed that CSObs cultured on enzyme-treated ECMs produced by CIObs responded with increased early *DCN* and *BGN* expression compared to cells on untreated ECMs. The lack of significance in *BGN* expression from cells on CSObs ECM could be attributed to greater concentrations of biglycan within these ECMs. These results confirm differences in the amount of DCN and BGN present in the enzyme-treated ECM in comparison to the untreated ECMs from both cell types.

To verify if the enzyme-modulated ECMs would exhibit impaired protein binding and resultant decreases in osteogenic markers in CSObs, we calculated the binding affinity of TGF-β1 and BMP-2 on the enzyme-treated ECMs. As expected, enzyme-treated ECMs had significantly higher K_d_ values (reduced binding) for both proteins compared to the untreated groups. TGF-β1 stimulates proliferation of osteoprogenitor cells and inhibits differentiation of mature osteoblasts.[34] BMP-2 is a potent inducer of osteoblastic differentiation. By stripping the ECMs of binding sites for these proteins, it may be possible to impair osteoblastic activity of CSObs. These data support this hypothesis, as we did not detect significant increases in expression of mRNA for *ALP*, *RUNX2*, or *COL1A* for cells cultured on enzyme-treated ECMs in the presence of TGF-β1. Moreover, CSObs cultured on enzyme-treated ECMs in the presence of exogenous BMP-2 consistently exhibited reduced expression of these three markers compared to cells on untreated ECMs.

These data demonstrate that binding of osteoinductive proteins to underlying cell-secreted ECMs and resultant osteogenic signaling is distinct between patients diagnosed with premature cranial fusion and healthy controls. These differences are attributed to the composition and activity of ECM constituents such as proteoglycans and not simply due to the presence of highly hydrophilic hydroxyapatite deposited by active osteoblasts. Collectively, these data suggest that the increased osteogenic potential of osteoblasts from craniosynostosis patients is at least partially derived from differences in the local presentation of endogenous osteoinductive proteins from the surrounding ECM. The ECM may serve as a means for the local osteogenic cells to modulate the effects of the various growth factors. While a more thorough characterization of the ECM protein-cytokine interaction is necessary to completely understand the signaling mechanism in these cells, the use of enzyme treatment provides an additional tool to reduce the binding of endogenous BMP-2 binding which may be useful in reducing osteoblast activity of native cells.

## Materials and Methods

### Ethics statement

All specimens were obtained following informed written consent according to an approved protocol granted by the Institution Review Board at the University of California, Davis. All patients with coronal craniosynostosis were clinically assessed and found to have nonsyndromic coronal craniosynostosis without associated extracranial congenital anomalies or developmental delays.

### Cell culture and ECM production

Osteoblasts were isolated from bone fragments following suturectomy as treatment for nonsyndromic craniosynostosis (CSObs), while normal calvarial osteoblasts (CIObs) were isolated from cranial bones of children undergoing surgical intervention for head trauma. Genetic analysis excluded mutations associated with syndromic forms of craniosynostosis in the relevant exons of *FGFR1*, *FGFR2*, *FGFR3* and *TWIST* genes as described by Boyadjiev *et al.* [2] Osteoblasts were maintained in Dulbecco’s modified eagle’s medium (DMEM, Invitrogen) containing 10% fetal bovine serum (FBS, JR Scientific) and 1% penicillin-streptomycin (Mediatech). Cells were used at passages 4-6. At confluence, cells were trypsinized and seeded in 24 well plates at 30,000 cells/cm^2^ and cultured in maintenance media (DMEM/10% FBS). After 24 h, the media was replaced with osteogenic media (maintenance media containing 10 nM dexamethasone, 50 µg/ml ascorbate-2-phosphate, and 10 mM β-glycerophosphate, all from Sigma), and the cells were cultured for another 5 d. The cells were cultured in 5% or 21% oxygen as previously described.[35] After 5 d, osteoblast-secreted ECMs were decellularized by incubation in phosphate buffered saline (PBS) containing 20 mM NH_4_OH and 0.5% (w/v) Triton-X for 15 min at 37°C.[14] ECMs were treated with PBS containing DNase (100 units/ml; Sigma) for 1 h, and resulting ECMs were then rinsed with PBS, air dried, and used immediately for subsequent experiments. Total protein concentrations in the ECMs were measured using Amido black staining.[36]

### Adsorption of growth factors to osteoblast-secreted ECMs

Recombinant human TGF-β1 and BMP-2 (Peprotech) were reconstituted to a final concentration of 1 mg/ml in PBS containing bovine serum albumin (BSA) as a carrier. The growth factors were fluorescently labeled using the Dylight 488 labeling kit (Pierce Biotechnology) as described by the manufacturer. Briefly, the growth factors were incubated with the fluorescent dye for 1 h at room temperature. The unincorporated dye was removed by passing the mix through a desalting spin column. The labeled growth factors were diluted in PBS to obtain a range of concentrations from 0 to 200 ng/ml. ECMs prepared as described above were incubated overnight at 4°C with 200 µl of PBS containing either TGF-β1 or BMP-2 at different concentrations. After 24 h, the growth factor solution was collected, ECMs were rinsed twice with PBS to remove unbound growth factor, and the distribution of bound protein on ECMs was imaged using a Nikon Eclipse TE2000-U fluorescent microscope. Growth factor-bound ECMs were then scraped in PBS (100 µl/well) using a cell scraper, and bound concentrations of each protein were quantified *via* fluorescence (excitation: 493 nm, emission: 518 nm) with a plate reader (Synergy HTTR, Biotek). Growth factor concentration was calculated from a standard curve corrected for BSA. All concentrations were normalized to ECM protein concentrations, and the dissociation constants (K_d_) were calculated from a Scatchard plot analysis using Prism (GraphPad Software, Inc.). [37]

### Enzymatic treatment of ECMs

Osteoblasts were seeded in 24 well plates at 30,000 cells/cm^2^ in maintenance media. After 24 h, the media was changed to serum-free osteogenic media containing heparanase (heparanase I, II, III; 1.2 mU/ml) and/or chondroitinase ABC (50 mU/ml, both from Sigma) to disrupt GAG formation. The cells were cultured for an additional 5 d with the medium changed every 3 d. ECMs deposited by cells without enzyme treatment served as controls. The ECMs were then decellularized as described above. Binding constants for TGF-β1 and BMP-2 on enzyme-treated ECMs were quantified by Scatchard plot analysis.

ECMs were reseeded with CSObs or CIObs at 30,000 cells/cm^2^ and cultured in 5% O_2_ and osteogenic media for up to 10 d. The expression of osteogenic markers including alkaline phosphatase (*ALP*), runt-related transcription factor 2 (*RUNX2*), and collagen-Ia (*COL1A*) was evaluated using qPCR.[38] Briefly, ECMs were rinsed with PBS and total RNA was collected using the RNeasy Mini kit (Qiagen). 300 ng of total RNA was reverse-transcribed with Superscript II Reverse Transcriptase (Invitrogen). qPCR was performed using the TaqMan1 Universal PCR Master Mix (Applied Biosystems) on a Mastercycler1 Realplex2 (Eppendorf). Primers for *ALP*, *COL1A*, *RUNX2*, decorin (*DCN*), biglycan (*BGN*), and *RPL13* were purchased from Applied Biosystems. Amplification conditions were 50°C for 2 min, 95°C for 10 min, followed by 40 cycles at 95°C for 15 s and 60°C for 1 min. Quantitative gene expression values were normalized to *RPL13* and presented as ΔC_t_ values calculated as fold change in gene expression with respect to expression of the housekeeping gene. Mineralization was quantified by measuring calcium deposition as in indicator of late stage osteoblastic differentiation. Osteoblasts seeded on ECMs were collected in 0.9N sulfuric acid and incubated at 37°C overnight. Non-cell seeded ECMs were considered as background to account for any calcium present in the ECMs. Calcium content was quantitatively assessed using the OCPC calorimetric method.[39] Calcium deposited by cells after culturing on the different ECMs was calculated by subtracting the background from total calcium levels.

We further studied the expression of osteogenic markers by osteoblasts seeded on enzymatically-treated ECMs when supplemented with exogenous TGF-β1 or BMP-2. After decellularization, enzyme-treated ECMs produced by CIObs were seeded with CSObs at 30,000 cells/cm^2^ and cultured in serum-free osteogenic media containing either 10 ng/ml TGF-ß1 or 100 ng/ml BMP-2. CSObs seeded on non-enzyme treated CIObs-produced ECMs or directly on tissue culture plastic (TCP) and maintained in growth factor-supplemented media served as controls.

### Characterization of ECMs

#### Staining for GAG and mineral content

Enzyme treated ECMs were prepared as described above in the presence or absence of disrupting enzymes. GAG content within ECMs was quantified using an Alcian blue stain.[40] Briefly, ECMs were rinsed in distilled H_2_O, and then incubated overnight in 0.1 N HCl containing 0.1% Alcian blue (Sigma). The ECMs were rinsed three times in distilled H_2_O, extracted using 200 µl of 0.1N HCl, and absorbance was measured at 620 nm. The presence of mineral in enzyme-treated and native ECMs was qualitatively assessed using Alizarin red staining.[41]

#### Immunohistochemical staining of ECMs to observe composition

The presence of collagen 1, BGN, syndecan-2 (SYN-2), and fibronectin (FN) in ECMs was examined using immunohistochemical staining with human primary antibodies (Santa Cruz Biotechnology) performed with a mouse specific HRP/DAB detection kit (Abcam) following the manufacturer’s instructions. Briefly, ECMs were blocked with 200 µl/well of H_2_O_2_ for 10 min, followed by three rinses with wash buffer (10% TBST, 1% Tween). ECMs were further blocked in 200 µl/well of protein block to prevent nonspecific protein binding. ECMs were then incubated overnight at 4°C with specific primary antibodies (1:50 dilution in PBS containing 2% (v/v) protein block). Controls were incubated in buffer solution. After rinsing, biotinylated goat anti-mouse IgG was applied for 30 min at room temperature, ECMs were rinsed, and streptavidin peroxidase was applied for 10 min. After rinsing, 200 µl of the DAB chromogen-substrate complex (20 µl chromogen to 1 ml substrate) was applied to each well for 10 min. The ECMs were rinsed in tap water 7-8 times, air dried, and imaged using a Nikon Eclipse TE2000-U microscope.

### Statistical analysis

Results are expressed as mean ± standard deviation of the mean. All assays were performed in triplicate unless otherwise mentioned. Statistical significance was determined using the Bonferroni post-*hoc* test and probability values (*p*) < 0.05 were considered significant.
